# Exploring High-Precision Non-Assembly Mechanisms: Design of a Vitrectome Mechanism for Eye Surgery

**DOI:** 10.3390/ma16051772

**Published:** 2023-02-21

**Authors:** Kirsten Lussenburg, Marta Scali, Maarten Stolk, Daisy Robijns, Aimée Sakes, Paul Breedveld

**Affiliations:** 1 Bio-Inspired Technology Group (BITE), Department BioMechanical Engineering, Faculty of Mechanical, Maritime, and Materials Engineering, Delft University of Technology, 2628 CD Delft, The Netherlands; 2 Dutch Ophthalmic Research Center International (DORC), 3214 VC Zuidland, The Netherlands

**Keywords:** additive manufacturing, PolyJet, non-assembly, eye surgery, multi-material, high-precision

## Abstract

A vitrectome is a commonly used instrument in eye surgery, which is used to cut and aspirate the vitreous body out of the eye. The mechanism of the vitrectome consists of miniature components that need to be assembled by hand due to their size. Non-assembly 3D printing, in which fully functional mechanisms can be produced in a single production step, can help create a more streamlined production process. We propose a vitrectome design based on a dual-diaphragm mechanism, which can be produced with minimal assembly steps using PolyJet printing. Two different diaphragm designs were tested to fulfill the requirements of the mechanism: a homogenous design based on ‘digital’ materials and a design using an ortho-planar spring. Both designs were able to fulfill the required displacement for the mechanism of 0.8 mm, as well as cutting forces of at least 8 N. The requirements for the cutting speed of the mechanism of 8000 RPM were not fulfilled by both designs, since the viscoelastic nature of the PolyJet materials resulted in a slow response time. The proposed mechanism does show promise to be used in vitrectomy; however, we suggest that more research into different design directions is required.

## 1. Introduction

**Figure 1:**
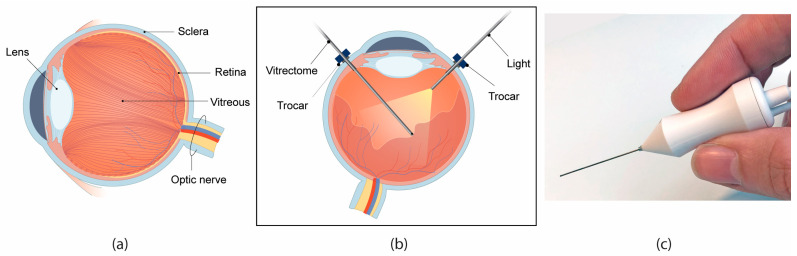
Vitrectomy surgery. (**a**) Schematic cross-section of the anatomy of the eye, showing the orientation of the collagen fibre network in the eye, based on [3]. (**b**) Schematic of the set-up during a typical vitrectomy. (**c**) An example of a commercially available vitrectome as produced by D.O.R.C. (Dutch Ophthalmic Research Centre, Zuidland, The Netherlands).

The vitreous body is a gel-like substance that fills the inside of the eye, consisting of water (99%) and a collagen fibre network intermixed with hyaluron (Figure 1a) [1]. Its main function is to stabilize the eye and absorb shocks from movement or mechanical impact that can reach the retina or lens [2]. The collagen fibres are connected to internal structures of the eye, such as the retina, creating a more dense network towards the outer edges of the eye [3]. In certain operations in the posterior segment of the eye, it is necessary to remove part or all of the vitreous, for instance to gain a clear path of access to the retina, without applying traction to the delicate internal structures. For this purpose, a vitrectome or vitreous cutter is used, which simultaneously cuts the vitreous in smaller pieces and aspirates it through the instrument out of the eye (Figure 1b,c). Such an instrument generally consists of a small hollow knife, which is placed inside the eye, with a driving and aspiration mechanism inside the handle, which remains outside of the eye. 

The vitrectome, as well as other instruments commonly used in ophthalmology, consists of very small components with strict requirements regarding their precision and alignment. To produce these type of devices, manual assembly is still the most feasible solution; however, this is time-consuming, expensive, and susceptible to human errors [4]. Automatic assembly processes are generally costly to implement in the manufacture of small and intricate surgical instruments, such as a vitrectome, as they ask for highly advanced robotized assembly systems that would require high investments for medium-scale manufacture [4]. As an alternative, the use of additive manufacturing (AM) or 3D printing offers potential to streamline the production process, since its potential for high geometrical complexity allows for a high integration between parts with different functionalities, thereby reducing the need for assembling multiple parts [5]. AM can contribute to the optimization of the production and assembly chain for medical devices, since fully functional mechanisms can be produced in a single production step, without needing to be assembled [5,6]. These so-called non-assembly mechanisms make production cheaper and easier, reducing the need for specialized knowledge for the assembly and fine-tuning of the mechanism. 

Non-assembly designs have successfully been explored for the design of advanced, steerable end-effectors for minimally invasive instruments [7,8,9,10], as well as deployable implants [11,12], using different types of AM technology. To date, there have been only few attempts towards 3D printing instruments for eye surgery. Some examples are miniature 3D printed trocars [13,14] and a surgical puncturing needle, which can puncture a retinal vein with a programmable stroke length [15]. The difficulty in creating non-assembly, 3D printed instruments for eye surgery is that these instruments require specific, precise functionalities, in a relatively small design space. This requires a high level of integration between components, which effectively means a new design has to be created specifically for AM, in which both functionality and manufacturability are taken into account [16]. In this study we explore the use of AM for the design and development of a non-assembly vitrectome mechanism used in eye surgery, with the intention of simplifying its fabrication and assembly process to facilitate its use as a low-cost disposable product. Special attention is paid to attempting to fulfill the specific requirements of the vitrectome to make it to be feasible to be used in eye surgery. 

## 2. Design and Fabrication 

### 2.1. Specifications

**Figure 2:**
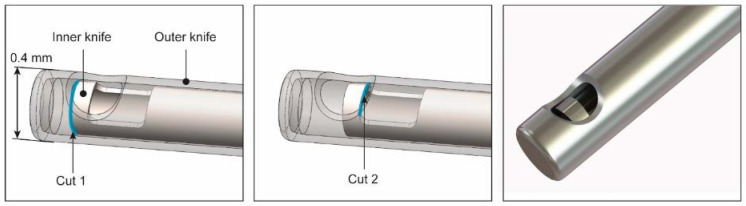
Close-up of the cutting principle of a vitrectome. The inner knife moves with a linear cutting motion within the stationary outer knife. Both knives have an opening on the side called an aspiration port, these two ports cause the inner knife to create two cuts instead of one per reciprocating motion (the cutting sides of the inner knife are highlighted with a blue line).

A vitrectome generally consists of two hollow tubular knives and a driving mechanism. The outer knife is stationary and has a closed, blunt tip, with an aspiration opening (port) on the side. The driving mechanism moves the inner knife within the outer knife, with a guillotine-like linear cutting motion to cut the vitreous. A linear cutting motion of the inner knife is preferred over a rotary motion to prevent traction of vitreous that is pulled between the shearing blades [17]. Modern vitrectomes have both an aspiration port in the outer knife, as well as in the inner knife, to double the effectiveness of the cut (Figure 2). The end of the inner knife is connected to an aspiration system, through which the vitreous is removed from the eye. Sizes of outer knives currently used in eye surgery vary between 23G (0.65 mm) and 27G (0.4 mm) [18]. 

The mechanism that drives the cutting motion of the knives is located in the handle. Although different driving mechanisms are used in vitrectomes currently on the market, the most common drive systems are powered by an external pneumatic system [19], which is operated during surgery by means of a foot pedal. The system generates short pressure pulses, causing a linear, vibrating motion of the inner knife. Vitrectomes on the market today have a pulse rate of the driving mechanism that varies between 0 and 8000 pulses per minute, which results in 16,000 cuts per minute when two aspiration ports are used (as illustrated in Figure 2) [18]. A low cut rate is used for operations such as retinal shaving, where precision and control are important, whereas a high cut rate can be used to remove the central part of the vitreous. High cut rates result in less traction on the retina and are also associated with better removal of the vitreous, resulting in a shorter duration of the surgery [20,21]. 

The stroke length of the driving mechanism is based on the geometries and tolerances of the aspiration ports, with a safety factor to ensure that the vitrectome always fully opens both aspiration ports. The cutting force generated by the driving mechanism should be approximately 8 N in the forward and backward direction. Since the force to cut the actual vitreous is very low, the majority of the cutting force is required to overcome the friction between the knives and within the driving mechanism. The handle of the vitrectome needs to be small enough for operation by a one-handed precision-grip by the surgeon while enabling rotation between the fingers to position the aspiration port of the outer knife in different orientations. 

### 2.2. Driving Mechanism 

#### 2.2.1. Design

**Figure 3:**
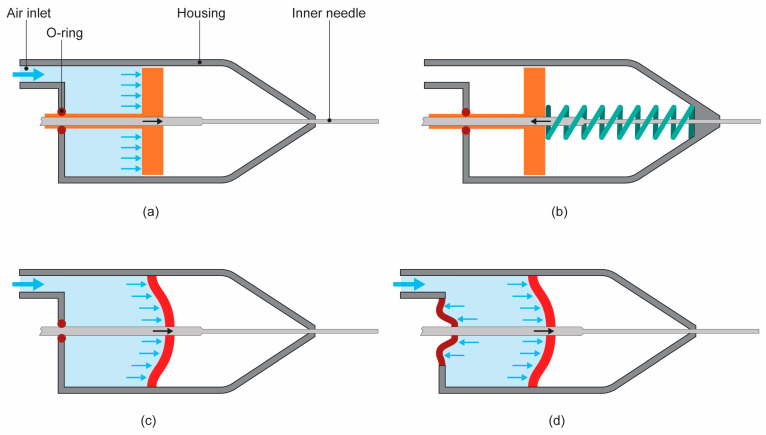
Evolution of the design for the non-assembly vitrectome driving mechanism. (**a**) Schematic design of a simple, pneumatically actuated driving system with a piston (orange) delivering a forward motion for the inner needle by means of air pressure. (**b**) Same design in which the backward force is delivered by a spring (green). (**c**) Piston–spring system replaced by a flexible diaphragm, which fulfills both the function of the piston and the spring. (**d**) Dual diaphragm system in which the back of the instrument is also sealed with a diaphragm.

In order to design a non-assembly vitrectome suitable for AM, we started from a simplified vitrectome design, which we adjusted for the different functions of the vitrectome and the manufacturing requirements of AM. A simple design for a pneumatically actuated translating system is a piston, shown implemented in a vitrectome in Figure 3a. The piston moves forward and backward when air pressure is applied to an airtight chamber on either side of the piston. Since most pneumatic systems used in vitreoretinal surgery only supply a positive pressure pulse, the vitrectome should either contain two air inlets with regulated valves, one on each side of the piston, or the mechanism should be designed in such a way that the return stroke does not require pressure. To keep the design as simple as possible, a spring can be implemented to provide the return stroke (Figure 3b). 

Although the piston–spring design has the potential to fulfill the functional requirements of a vitrectome, there are some problems using AM to create such a system. First, sliding surfaces are problematic for 3D printed parts, due to the presence of visible layer lines in the part, often called the staircase effect. This can hinder the sliding motion of the piston or create unacceptably high friction forces. Second, very tiny clearances need to be present between the piston and the walls of the housing to allow the piston to move while keeping an airtight chamber. Since the goal is to create a non-assembly mechanism, these clearances need to be incorporated within one 3D printed part. Small clearances printed in such a way run the risk of fusing of parts, while large clearances will not create an airtight chamber. To solve this, the piston–spring design was replaced by a flexible diaphragm, which can theoretically fulfill the function of both the piston and the spring, as shown in Figure 3c. The advantages of a diaphragm mechanism are that it is easier to print, since the parts are attached to each other, the diaphragm creates an effective seal for the air chamber, and a separate spring is no longer required. 

Since the inner knife needs to be connected to an aspiration system at the distal end, the knife needs to extend beyond the driving mechanism. This exit needs to remain airtight as well as to allow the inner knife to move, which can conventionally be solved by using a rubber O-ring. This effectively creates the same manufacturing problems as described above for the piston. As a solution, a diaphragm was implemented as well to both provide an airtight seal and allow movement of the inner knife (Figure 3d). The resulting design can be described as a dual diaphragm actuator. The additional advantage of this design is that it obtains a linear guidance for the inner knife, without the high required precision that is difficult to achieve using AM. 

#### 2.2.2. Working Principle

**Figure 4:**
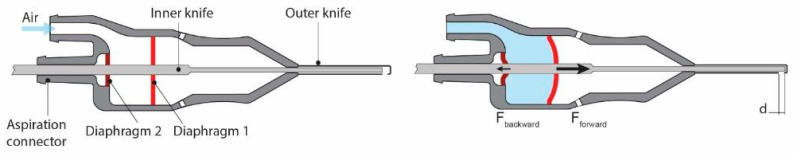
Working principle of the dual diaphragm mechanism. Left: schematic dual diaphragm design showing the various components of the design before air pressure is applied. Right: air pressure is applied to the chamber, causing both diaphragms to exert an opposite force on the inner knife. The larger diameter of Diaphragm 1 results in a net forward force, causing a displacement “d” of the inner knife.

The two diaphragms form an enclosed chamber, to which the pneumatic pressure pulse is applied. The inner knife is attached to the center of the diaphragms. When a pressure pulse is applied, the diaphragms are forced outwards, thus applying an opposite force on the needle. For two diaphragms of equal size, the force on the inner knife is equal in both directions, resulting in zero motion. In order to create a resulting force to move the inner knife in one direction, the diaphragms should have a different surface area. For the net force to point to the right at a positive pressure in the air chamber in Figure 4, Diaphragm 1 should have a larger surface area than Diaphragm 2. The net force developed by the mechanism in the forward direction (*F_net_*) is equal to the difference between the force applied to the needle by the large (*F_forward_*) and small (*F_backward_*) diaphragms:

*F_net_* = *F_forward_* − *F_backward_* (1)

The large diaphragm (Diaphragm 1) has multiple functions: (1) it converts the applied pressure to a forward motion of the inner knife; (2) it stores the spring energy required for exerting a force and movement in the backward direction; (3) it encloses the air chamber by forming a seal; (4) it holds the inner knife in the central position. The small diaphragm in the back of the instrument (Diaphragm 2) only functions as a seal that allows translation of the inner knife and keeps it centered. 

### 2.3. 3D Print Technology

Considering the design of the driving mechanism, the used AM technology should be able to produce both rigid and flexible structures in the same production step. Although for some materials flexibility can be created by producing a thin, slender structure, this depends on the minimum feature thickness that can be produced by an AM technology. For the small size of the vitrectome, it does not appear to be feasible to produce a diaphragm with a sufficient slenderness ratio. Another strategy is to combine stiff and flexible materials in one 3D printing step. Material jetting, often referred to as PolyJet printing (Stratasys Ltd., Valencia, CA, USA), is capable of depositing two or more different materials on a pixel-by-pixel level, referred to as a ‘digital material’ [22], which allows for the creation of monolithic, multimaterial structures in a single processing step. Therefore, this process was chosen to produce the non-assembly vitrectome. 

In the PolyJet process, an ink-jet print head moves in the horizontal xy-plane above the build platform. The ink-jet print head accurately deposits photopolymeric resin droplets on the build platform, which are instantly cured by ultraviolet (UV) light. The ink-jet print head has multiple nozzles that deposit different types of material in the same print, including a support material. Although the advantage of the process is that it can combine rigid and flexible materials in a single printing step, the behavior of these materials is difficult to predict, since they are heavily influenced by the settings of the 3D printer [23], such as the orientation on the build plate [24,25], UV exposure during printing [24,26], and the presence of support material [27]. In addition, the materials used in the PolyJet process exhibit viscoelastic behavior [27] and are both frequency- and temperature dependent [28]. Based on these factors, it is difficult to predict the final behavior of a mechanism in advance. 

### 2.4. Non-Assembly Vitrectome Design 

#### 2.4.1. Knives

Although the PolyJet process is able to produce small features with high resolution, it is not possible to produce the knives with the required tolerances and size, or with sufficient stiffness. Therefore, the decision was made to use off-the-shelf knives (D.O.R.C., Zuidland, The Netherlands), which will be attached in the final mechanism. These particular knives require a stroke length of the driving mechanism of 0.8 mm. The inner knife is connected to the rigid carrier in the center of the diaphragms, while the outer knife is connected to the housing in the front and is stationary.

#### 2.4.2. Main Body and Housing

**Figure 5:**
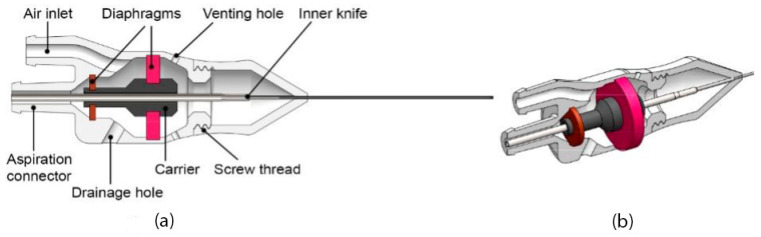
Design of the non-assembly vitrectome. (**a**) Cross-section showing the various components of the design. (**b**) Cross-section showing the full dual diaphragm mechanism. Diameter outer knife 0.4 mm, length outer knife 28 mm, length entire 3D printed vitrectome 80 mm.

Using PolyJet printing, the diaphragms can be produced in a flexible material, while the rest of the handle can be produced in a rigid material. The design of the diaphragms is experimentally explored in Section 3. The handle of the vitrectome should facilitate handling by the surgeon by means of a precision grip; therefore, we limited the outer diameter to 16 mm at its widest point, with a slight inward slope that allows for placement of the fingers. For sufficient rigidity, the handle was given a wall thickness of 1 mm. On the back of the handle are two connector ports, one to connect the aspiration channel to the hollow needle and the other one to connect the air chamber to the pneumatic system. To ensure that no pressure can build up outside the enclosed air chamber, two venting holes are located behind the large diaphragm to ensure this part of the vitrectome remains at atmospheric pressure. A rigid center portion, called the carrier in Figure 5a, is added to be able to hold the inner knife in the center of the diaphragms. The carrier is attached to both diaphragms, but not to the outer housing. 

In the PolyJet process, support material is always required for enclosed chambers [29], which means all internal cavities of the non-assembly vitrectome will be filled with support material. The support material is, however, water soluble and can be removed by soaking the part in water combined with mechanical removal, commonly by pressure washing with water. To provide sufficient access for the pressure washer, multiple drainage holes should be added to create a continuous path for the water [29]. The dual diaphragm design shown in Figure 5a,b contains two internal cavities: one between the two diaphragms and the other one between the large diaphragm and the tip of the instrument. For the first internal cavity, we opted to create a continuous channel by using the air inlet and adding one additional drainage hole of 2.5 mm diameter, as illustrated in Figure 5a. The drainage hole is closed off after removing the support to create an airtight cavity. 

For the second internal cavity between the large diaphragm and the tip of the instrument, in theory the venting holes can be used to remove the support material. However, since the knives cannot be printed, the carrier has to be accessible from the outside so that the inner knife can be mounted to it. To facilitate this, we added a screw connection in front of the large diaphragm, which is integrated into the two halves of the housing. Although this adds an assembly step to the design, screw thread is simple and quick to assemble without any tools. In addition, it will save time and effort in removing the support material. 

#### 2.4.3. Initial Prototype

**Figure 6:**
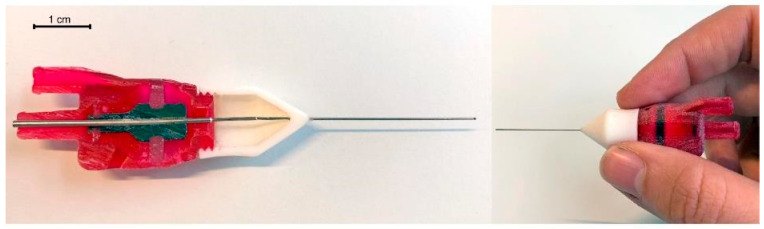
Initial prototype of the non-assembly vitrectome. **Left**: 3D printed model of the vitrectome cut in half, showing the two knives, the carrier in black and the diaphragms in light red. **Right**: 3D printed model of the vitrectome. The attachment of the outer diaphragm can be seen as a black line through the red housing. Diameter outer knife 0.4 mm, length outer knife 28 mm, length entire 3D printed vitrectome 80 mm.

A prototype of the vitrectome was 3D printed to test the clearances and whether support could be removed from all chambers. The prototype (Figure 6) was printed on an Objet260 Connex 3 (Stratasys, Ltd., Minnesota, USA), using Vero, a rigid material, Agilus30, a flexible material, and a support material, 706b. It was possible to remove most of the support material, although a thin layer remains on all surfaces, which is noticeable when scratching it with a fingernail. The outer knife was glued to the tip, and the inner knife to the carrier. The total prototype has a weight of 4.6 g.

## 3. Experimental Evaluation

### 3.1. Diaphragm Requirements

In this section, we explore the mechanical properties of the dual diaphragm mechanism. The mechanism has a number of specific requirements that need to be fulfilled by the diaphragms, as discussed in Section 3. The requirements of the large diaphragm are the following: (1) Provide a displacement of 0.8 mm; (2) Provide a forward force of 8 N as a result of the applied air pressure; (3) Provide a backward force of 8 N; (4) Obtain a cutting speed of ideally up to 8000 pulses per minute. Based on these requirements, the large diaphragm should be not only flexible enough to allow a forward motion with limited pressure but also stiff enough for a sufficient spring constant for a fast backward motion. For the small diaphragm, the only requirement is that it needs to be as flexible as possible while providing an airtight seal. When air pressure is applied, this diaphragm will generate a force in the opposite direction; therefore, this force should be minimized by means of a low stiffness and small surface area.

Because of the near-unlimited possibility of mixing materials in any ratio and any design and the lack of specific data on the material properties of PolyJet materials, we adopted an exploratory process, in which we tested different versions of the diaphragm to get a sense of the range of mechanical properties that the mechanism can obtain. The variables for these experiments are the design and material of the large diaphragm, while the rest of the mechanism is kept constant. 

### 3.2. Prototype Design and Production

**Figure 7:**
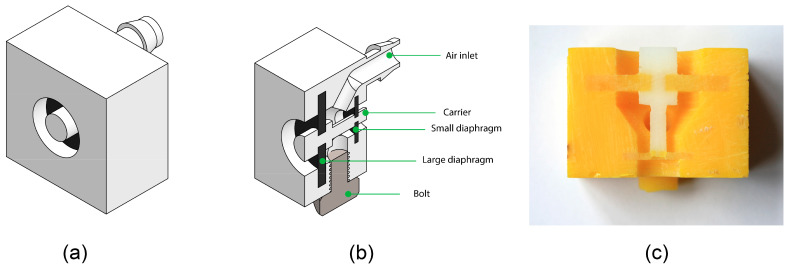
Prototypes of the dual diaphragm mechanism used for the experimental evaluation. (**a**) 3D model of the prototype. (**b**) Cross-section of the 3D model showing the various parts. (**c**) Cross-section of the 3D printed prototype.

A simplified prototype of the dual-diaphragm mechanism was designed for the tests (Figure 7a–c). The outer casing was converted to a rectangular shape to enable easy fixation of the prototype during the tests. The design of this prototype was kept constant, while we tested different versions of the large diaphragm. The prototypes were printed using an Objet260 Connex 3 multi-material printer, using Agilus30, a flexible material, and Vero, a rigid material. For the support of the inner cavity, the water-soluble support material 706b was used. To remove the support material, the samples were soaked in water for 2 days, after which the rest of the support was manually removed with a micro pressure washer. The drainage hole was sealed using a standard bolt after cleaning. The cutting knives were not included in this prototype. 

### 3.3. Experimental Design and Procedure

Initial experiments were conducted to test the proof-of-principle of the dual-diaphragm mechanism. First of all, the goal was to investigate whether a non-leaking air chamber could be printed and whether the sufficient displacement in the forward direction could be obtained. In addition, the forward cutting force, the backward cutting force, and cutting speed of the mechanism were determined. During the tests, measurements were performed on the carrier, which is the part of the mechanism that propels the inner knife. 

#### 3.3.1. Forward Cutting Force

**Figure 8:**
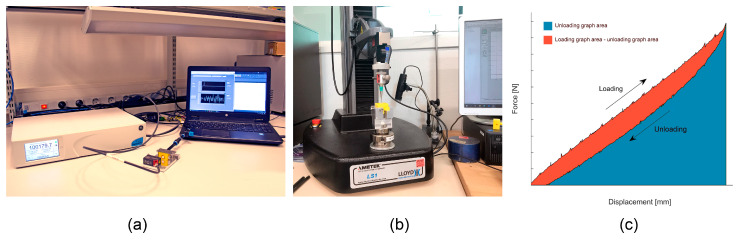
Set-ups used in the experimental evaluation. (**a**) Set-up for the forward cutting force test and cutting speed test. (**b**) Set-up for the backward cutting force test. (**c**) Example of a force displacement plot used to calculate the spring coefficients of the prototypes.

The first experiment measured the forward cutting force. This was tested by applying air pressure to the inner chamber and measuring the force generated by the mechanism without a displacement. A pressurized air supply (PACE 5000, General Electric Company, Boston, MA, USA) connected to a valve was used to apply a constant air pressure to the prototype. The prototypes were horizontally clamped in a custom aluminum platform to which a mini load cell (Futek, Irvinse, CA, USA) was mounted in contact with the carrier and connected to an analog signal conditioner (Figure 8a). First, a baseline pressure was determined per prototype by testing the response on a range of different pressure levels. Then, to determine the forward force, fixed levels of pressure were stepwise applied for a duration of 20 s, causing the carrier to exert a force on the load cell, after which the pressure was relieved. Whenever possible, three different prototypes were tested at least three times, unless a prototype ruptured during the tests.

#### 3.3.2. Backward Cutting Force and Spring Coefficient

The backward cutting force was measured using a tensile tester (Lloyd LS1EH, AMETEK STC, Berwyn, PA, USA.) with an attached 50 N load cell (AMETEK STC, Berwyn, PA, USA.). The prototype was clamped vertically to the base of the tensile tester, and a blunt needle was attached to the load cell, which was used to displace the carrier of the prototype from the outside (Figure 8b). The tensile tester was set to displace the carrier downwards by 0.9 mm with a speed of 0.6 mm/min. After reaching the maximum displacement, the tensile tester moved upwards while the load cell registered the force exerted by the carrier. The tests were repeated three times per prototype from the front and three times from the back. The measured data was plotted in a force-displacement plot (an example is given in Figure 8c. The slope of the loading curve of each measurement was used to determine the spring coefficient of the diaphragm. 

#### 3.3.3. Cutting Speed

A similar set-up as in the forward cutting force test was used to measure the cutting speed, except in this case a laser sensor (ILD1420-10, Micro-Epsilon, Ortenburg, Germany) was used to measure the displacement of the carrier. Again, the baseline pressure level was determined per prototype and applied in pulses of different pressure levels. The displacement of the carrier and the time required to reach this displacement were recorded from the moment a constant pressure level was applied. When the pressure was lifted, the displacement and time were recorded until the carrier had returned to its base position. This resulted in a response time for the forward motion as a result of the pressure and a response time for the backward motion as a result of the spring constant of the diaphragm. Whenever possible, three different prototypes were tested at least three times, unless a prototype ruptured during the tests.

### 3.4. Digital Material Diaphragms

#### 3.4.1. Design

The first design that was explored was a diaphragm made of a homogenous material with a simple disk-geometry, in which the material properties are responsible for the functioning of the mechanism. This design direction was used to determine the base characteristics, such as the displacement, force required for cutting, and speed, of a dual-diaphragm mechanism. Based on design guidelines for the PolyJet process, a “safe” thickness for a self-supporting wall is 1 mm [30]. Since the small diaphragm should have less stiffness, it was given this thickness of 1 mm. The outer diameter of the small diaphragm was set to 7 mm. The outer diameter of the large diaphragm was set to 14 mm, which is the maximum size based on the diameter of the housing and given a thickness of 2.3 mm. The carrier has a diameter of 6.5 mm in the large diaphragm and 3 mm in the small diaphragm. We tested three different digital material mixtures: (1) a flexible version of 20% Vero and 80% Agilus30, (2) a medium version of 50% Vero and 50% Agilus30, and (3) a stiff version of 80% Vero and 20% Agilus30, as indicated in Table 1.

#### 3.4.2. Results 

**Figure 9:**
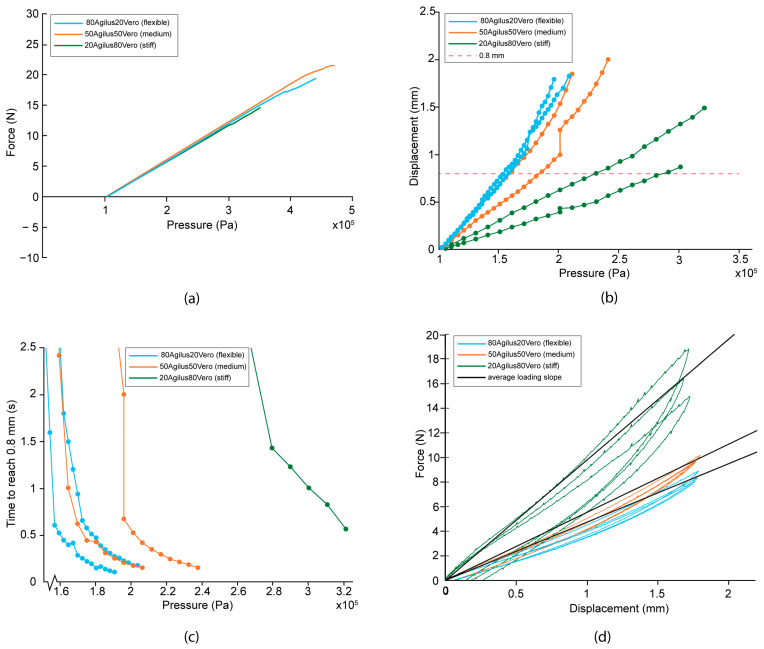
Test results of the digital material diaphragms. (**a**) The average forward force as a function of the pressure. (**b**) The displacement of the carrier as a function of the pressure. Only two prototypes for each material could be tested due to failure. (**c**) The time it took the carrier to reach a forward displacement of 0.8 mm as a function of different pressure levels. Only two prototypes for each material could be tested due to failure for the medium and stiff materials. Only one of the stiff prototypes (20Agilus80Vero) is plotted, and the other prototype failed during the test. (**d**) The loading and unloading curves of the prototypes, illustrating the hysteresis present in the mechanism.

All of the tested digital material prototypes were able to reach a minimum displacement of 0.8 mm. The backward cutting force, spring coefficient, and pressure to reach the displacement per prototype are summarized in Table 2. Figure 9a shows the average maximum measured forward force generated by the digital material diaphragms on different pressure levels. The graph shows that the forward force output of all the tested prototypes is nearly identical. All prototypes were able to reach a forward force of more than 8 N when an air pressure of 250 kPa was applied. 

The relationship between the applied pressure and the forward displacement of the carrier is fairly linear, as shown in Figure 9b. The stiff prototypes (20Agilus80Vero) required a higher pressure to move the carrier than the flexible material (80Agilus20Vero), as expected. In Figure 9c, it can be seen that a higher pressure also resulted in a faster response for each prototype. However, the stiff prototypes did not have a faster response than the flexible prototypes. The flexible prototypes were more responsive for both the forward and backward movement than the medium or stiff prototypes. The fastest time recorded in the tests was 0.11 s in the forward motion for the flexible prototype, before the diaphragm failed. 

Figure 9d shows the average force-displacement curves for the prototypes. The loading part of the cycle corresponds to the force required to displace the carrier, which is fairly linear in all cases. The unloading part of the cycle is the backward force generated by the diaphragms. The stiff prototypes reached the highest backward cutting force of 8.9 N on average at a deflection of 0.8 mm, while the medium and flexible prototypes showed values of 3–4 N on average. The calculated spring coefficients of the diaphragms are given in Table 2 and are in a range between 4.5 N/mm for the most flexible prototypes and 11.7 N/mm for the stiffest prototypes. These spring coefficients show that the stiffness of the mechanism does not increase linearly with the percentage of Vero. The difference between the loading and the unloading curves indicates hysteresis in the system, which is negative for the efficiency of the design. This results in a longer time for the diaphragm to return to its initial position, as well as a lower backward cutting force. The results suggests that Agilus30 is more capable of storing and releasing energy than Vero, as the prototypes with the highest percentage of Agilus30 have the smallest average hysteresis area. 

The results obtained in these first experiments show that it is possible to create a dual diaphragm mechanism using PolyJet printing, with the required displacement and forward force. However, the backward force and cutting speed do not yet fulfill the requirements. We speculate that this can be attributed to the viscoelastic nature of the digital materials. Therefore, we designed and tested another version of the large diaphragm in which the functions of the diaphragm are separated from the materials. 

### 3.5. Spring Reinforced Diaphragms

#### 3.5.1. Design

**Figure 10:**
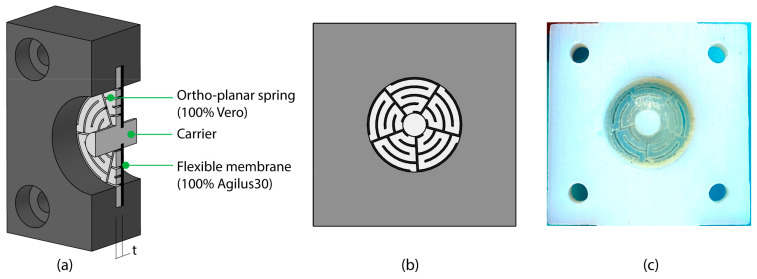
Design of the spring-reinforced diaphragms. (**a**) 3D model showing the cross-section of the prototype, with a flexible membrane printed in 100% Agilus30 and on top an ortho-planar spring printed in 100% Vero. (**b**) Top view of the spring design. (**c**) Photograph showing the printed prototype.

The second design was aimed at improving the speed characteristics of the mechanism. For this design, we separated the function of storing spring energy for the backward motion and the function of airtight enclosing of the air chamber. In this case, a flexible membrane of 100% Agilus30 with a thickness of 0.5 mm is responsible for sealing the air chamber, while an ortho-planar spring printed directly on top in 100% Vero is responsible for the speed and backward motion (Figure 10a). Ortho-planar springs are a type of planar spring, which typically has a central platform connected to a base by means of flexible segments [31], allowing a linear, out-of-plane motion [32]. The design of the spring is shown in Figure 10b,c. The spring covers as much of the surface of the diaphragm as possible, in order to prevent the flexible membrane from being pushed through the gaps under air pressure, by using minimum clearances of 0.25 mm, in accordance with PolyJet design guidelines. 

The spring stiffness can be varied by changing the thickness of the ortho-planar spring printed on top of the diaphragm. To investigate the influence of the thickness, we used Finite Element Modelling (FEM) (SolidWorks, Dassault Systemes SolidWorks Corp., Waltham, MA, USA) to calculate different thicknesses, using the material properties of Vero as supplied by the manufacturer [22]. First, we calculated the thickness required for a completely solid diaphragm made of 100% Vero in order to deliver a backward force of 8 N, and this thickness was calculated to be 0.28 mm. Since the spring shape will be weaker than a completely solid diaphragm, we also calculated the required thickness of our spring design to deliver 8 N, which was 1.12 mm. Based on these values, we tested three different thicknesses of the spring, summarized in Table 3: one with the same thickness as a completely solid diaphragm (Z0.28), one with the calculated thickness to deliver 8 N (Z1.12), and one with double this thickness (Z2.24).

#### 3.5.2. Results

For the spring diaphragms, only the tests for the cutting speed, both forward and backward, and the backward cutting force were repeated. Table 4 summarizes the results of these tests. 

**Figure 11:**
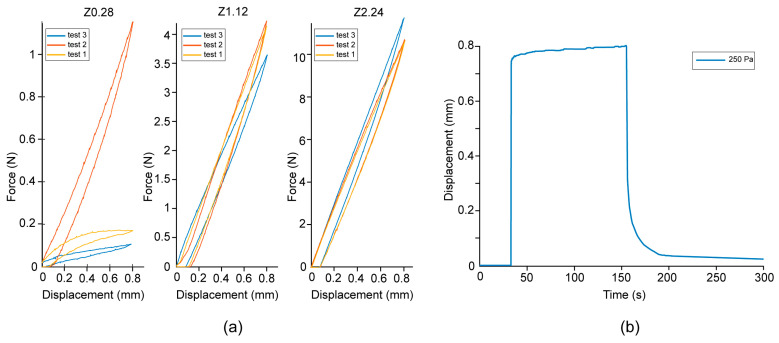
Results from the spring-reinforced diaphragm tests. (**a**) The force displacement curves for the three different prototypes: Z0.28 (left), Z1.12 (middle), and Z2.24 (right). Note the different scaling of the *y*-axis. (**b**) Example of a time-displacement curve for the Z1.12 prototype at 250 Pa pressure.

Table 4 and Figure 11a illustrate that the thicker springs show a higher backward force at 0.8 mm displacement. However, the results do not correspond with the FEM calculations. The Z1.12 design was expected to show a force of 8 N, but instead shows only 4 N. The Z0.28 prototypes show a large deviation between tests, which could indicate that the prototypes were not printed fully airtight. The Z2.28 prototype is the only design that can fulfill the requirement of 8 N. The results show that a thicker spring leads to a higher spring coefficient; however, this does not scale linearly. 

For the forward speed, the fastest response times for the entire 0.8 mm displacement are given in Table 4. The Z0.28 prototype did not reach an extension of 0.8 mm before breaking. For the Z1.12 and Z2.24 prototypes, only one test could be performed before the prototypes broke, at pressures of 270 Pa and 600 Pa, respectively. For the backward motion, the speed for the entire 0.8 mm could only be measured for the Z1.12 prototypes, since the other prototypes were not able to make the entire displacement due to breakage or plastic deformation. In general, the results from the cutting speed test show extremely large deviations between the results, more than was seen with the digital material diaphragms. 

Figure 11b shows an example of the time-displacement graph of the Z1.12 design of the entire movement of the diaphragm, in which the forward motion is a result from the applied pressure, and the backward motion is a result of the spring force of the diaphragm. The initial forward displacement as a result of the pressure up to approximately 0.7 mm happens rapidly, after which the speed stagnates for the final 0.1 mm displacement. An explanation for this might be viscoelastic creep within the material [33]. Comparable curves are obtained for all prototypes. Examining these curves shows that within the first 0.4 mm of the forward motion, all prototypes exhibit linear behavior and are able to reach a displacement of 0.4 mm within 0.01 s. The fastest response times are given in Table 5. 

Similarly, the initial backward response of the diaphragm as a result of the spring force of the diaphragm happens rapidly, with a stagnation in response time at around 0.2 mm. Table 5 shows the fastest backward response times for the displacement between 0.6 mm and 0.2 mm. Although these responses are significantly faster than for the entire 0.8 mm displacement, the results show that increasing the stiffness of the spring does not increase the backward speed of the mechanism. 

## 4. Discussion 

### 4.1. Production

The goal of this study was to explore the possibility of creating a non-assembly vitrectome mechanism that is able to fulfill the specific requirements of the vitrectome. We have presented a design that was theoretically a non-assembly; however, in practice, it required some assembly steps due to the chosen AM technology and the need to include off-the-shelf knives. Without any assembly steps, the post-processing time would increase due to the difficulty of removing the support material, placing the knifes, and the need to seal multiple drainage holes after cleaning. The division into two parts using screw thread makes it easier to remove all the support material and to attach the knives, resulting in a shorter total production time. Since the screw thread is integrated into the printed part, the assembly is easy and straightforward, and no specific alignment of parts is required. The screw thread is located in the part of the vitrectome that is at atmospheric pressure; therefore, the air tightness of the connection is not of concern. For these type of designs, we suggest that rather than focusing on eliminating assembly altogether, the focus should be on creating ‘smart’ assembly solutions, which can reduce the overall production time and simplify the production process [34]. Future developments in AM technology, for instance in the area of printing truly different materials, such as combinations of polymers and metals, could lead to better applicability of completely non-assembly designs. 

The advantage of the PolyJet process is the ability to print with multiple materials in the same printing step, which provides opportunities for miniature, complex mechanisms, where there is less design space to influence the material behavior by geometry. Unfortunately, there is limited information available on how PolyJet materials behave, especially considering the near-limitless design possibilities of 3D printing, and therefore it is hard to predict how they will respond. In addition, the challenge of creating a high-precision mechanism using AM is that it is difficult to control all variables in order to get a reproducible result, as was evidenced by the breakage of multiple prototypes. This means still a lot of research is needed to be able to produce these kinds of mechanisms consistently and reliably. 

### 4.2. Performance

#### 4.2.1. Cutting Force

The initial tests with the digital material diaphragms showed that a forward force of at least 8 N can be created by the mechanism. The force was shown to be linearly related to the applied pressure. In theory, a higher cutting force can be obtained by means of a higher pressure, as long as the diaphragms are able to withstand this pressure. To obtain the required backward cutting force, a higher stiffness of the diaphragm is necessary, as was illustrated by both the digital material diaphragms and spring diaphragms. A higher percentage of Vero increases the stiffness of the mechanism for the digital material diaphragms; however, the stiffness of the mechanism does not increase linearly with the percentage of Vero. To obtain the required backward cutting force with the tested dimensions of the diaphragm, a material mixture of minimally 80% Vero is required. For the spring diaphragms, a thicker spring results in a stiffer diaphragm, although again this does not increase linearly. The spring diaphragm with a thickness of 2.24 mm showed a higher backward force than the stiffest digital material diaphragm with similar thickness, indicating that the used material has a significant influence on the backward force. 

#### 4.2.2. Cutting Speed

The cutting speed of the mechanism still poses a challenge. For the digital material diaphragms, the most flexible prototypes were more responsive for both the forward and backward motion than the medium or stiff prototypes. The time it took for each of the prototypes to reach a forward displacement of 0.8 mm depends on the applied air pressure; however, the diaphragms failed before a high enough pressure could be applied to reach the required response time. The stiffer prototypes required a higher pressure to move the carrier forward than the flexible prototypes. However, this did not result in a faster response time than the flexible prototypes, in both forward and backward directions they were slower. Therefore, it can be concluded that the Vero material has a negative influence on the movement speed of the mechanism. Although even the flexible prototypes were almost 15 times too slow as compared to the desired cutting speed, the stiff prototypes were 75 times too slow. The spring diaphragms performed worse in general. The forward speed was slower than for the digital material diaphragms. This was partially caused by the fragility of the spring diaphragms; therefore, less pressure could be applied before the prototypes broke. We observed no improvements in backward time, and multiple spring prototypes were not able to displace the entire 0.8 mm, due to breakage or permanent deformation. 

When examining the motion of the spring diaphragms further, it was noticeable that the last 0.2 mm of each movement contributed significantly more to the total cutting speed than the first part of the movement. When omitting the first and last 0.2 mm, a more realistic approximation of the speed of the mechanism is obtained. For the first 0.4 mm forward motion, the required time for this motion was lower than 0.01 s. A similar effect was seen for the backward response, although this still remains too slow to obtain the desired cutting speed. The test results show no clear relation between the thickness of the spring and the backward speed, since the fastest response was obtained by the thinnest spring. Regardless, these results indicate that there is potential to improve the cutting speed by allowing the mechanism a longer stroke length. More tests are necessary to investigate this effect and determine an appropriate stroke length. Once the required cutting speed is met, high-speed durability tests have to be carried out to investigate how long our 3D printed mechanism will last as compared to conventional vitrectome designs.

#### 4.2.3. Materials and Design

The viscoelastic behavior of the printing materials is clearly visible in the response times of the prototypes. Contradictory to our expectations, the stiffer diaphragm designs did not show a faster response, both when a stiffer material mixture was applied and when a thicker diaphragm was applied. It appears that Vero exhibits more hysteresis than Agilus30, which is visible in Figure 9d. This leads to two contradictory requirements: a stiffer diaphragm is necessary to obtain the required backward cutting force; however, the stiff diaphragms show a poor response time and cannot obtain the desired cutting speed. The influence of the material properties of PolyJet materials on the requirements cannot be disregarded and cannot be solved by design alterations alone. This illustrates that for precision mechanisms produced using PolyJet, more research is necessary into the response of these materials [33].

Between the two diaphragm designs, it seems that the digital material diaphragms performed somewhat better than the spring designs. The separation of the different functions of the large diaphragm does not appear to provide a benefit in the performance of the mechanism. FEM calculations were not able to predict the behavior of the springs; therefore, we can assume that other forces are at play within the material, or that the influence of the flexible membrane changes the behavior of the system. Another effect that might be at play is the connection between the flexible membrane and the rigid material of the spring, which could result in shear forces. The digital material prototypes were more robust and could withstand multiple rounds of testing; however, it should be tested whether they are able to withstand repeated cycles during actual use. 

As an alternative to 3D printing in resins, metal 3D printing could also be interesting to investigate. Although manufacturing the flexible diaphragms out of metal could potentially solve our issues with viscoelasticity, 3D printing in metal could also result in new challenges related to printability, surface roughness, brittleness, and resolution, leading to relatively high required wall thickness and high stiffness.

### 4.3. Limitations of the Tests

Breakage of the prototypes may have limited the accuracy of the test results. A leak was only noted after the diaphragm completely broke or when the diaphragm could not be moved forward sufficiently by pressure. Therefore, it is possible that a small leak in the diaphragm was present during testing, which might have influenced the speed of the forward motion. In addition, it was possible for the ortho-planar springs to break without leakage of the flexible membrane, which could only be noticed by visual inspection after performing the tests, without knowing at which point the breakage occurred. 

The test set-up only examined one single forward and backward motion. This does not match the behavior of the actual vitrectome during use. During the tests with air pressure, the pressure was applied until a complete extension was reached. In an actual use scenario, the external pressure supply system will give multiple short pressure pulses in short succession. This means that at the same pressure level, the diaphragm might not completely extend. Moreover, it is likely that the 3D printed mechanism will not have returned to its start position before the next pulse is given. More testing is necessary to determine the response of the mechanism in a more realistic use scenario, as well as to examine the influence of the time dependency of the materials in this scenario. 

The time dependency and viscoelasticity of the materials could also have influenced the test results. Since a new baseline pressure had to be determined for each different prototype, relaxation effects could have occurred due to the initial extension of the diaphragm. In addition, the duration of the tests differed per prototype, since the diaphragms were pressed forward until an extension of 0.8 mm was obtained, regardless of the time it took. These effects could also have occurred due to previous tests performed with the same prototype. Although we attempted to use new prototypes for each test, this was not always possible due to breakage of the prototypes. These effects could account for the deviations between the results of some of the tests. The inaccuracies of the tests also partly reflect the main problem of producing this design using PolyJet, showing fast breakage and a lack of consistency between prototypes. 

### 4.4. Future Design Directions

In the experiments, we have seen that the closer the displacement is to the zero-position of the diaphragm, the slower the movement of the diaphragm. We hypothesize that by shifting the displacement away from the zero-position by applying a pre-tension, we can obtain a faster return stroke. This way, the translation of the diaphragm will take place from for instance 0.2 mm to 1.0 mm, instead of from 0.0 mm to 0.8 mm. To accomplish this, a pre-displacement can be applied to the diaphragm in a mechanical or pneumatic way. Mechanically, this can be accomplished by integrating a ‘pillar’ in the top half of the vitrectome body, which will displace the carrier by a predetermined amount during assembly of the screw thread. An integrated 3D printed helical spring could serve both as displacement as well as aid in the backward motion and could therefore be a promising direction to explore further. Although preliminary tests showed that applying a pre-displacement has a positive effect on the backward response time, it may also reduce the lifespan of the diaphragm. More extensive testing should find an optimum balance between response time and lifespan. In addition, the effect of creep of this solution will need to be examined, as this may reduce the effect of the pre-tension over time. 

It became clear that the presence of the Vero material, although it has a higher stiffness, slows the response time. An alternative could be to only use the flexible material Agilus30 for the diaphragm, with a larger thickness to provide the necessary stiffness. However, this type of diaphragm runs the risk of having a short lifespan due to tensile stresses in the material [35]. Alternatively, a corrugated diaphragm design could be tested. Due to the limitations to the size of the vitrectome, it would be necessary to obtain a lower wall thickness to create corrugations within the available space in the vitrectome body. In addition, the proper material mixture for this type of design should be investigated. Both diaphragm designs that were tested in this research have a cross-section with a constant thickness; however, a variable thickness for the diaphragm can also be implemented. The largest deflection should be at the center of the diaphragm, and the smallest deflection on the edges; therefore, a variable thickness with the highest thickness on the edges could be investigated to adjust the stiffness of the diaphragm. 

To circumvent the slow backward response of the diaphragm, an alternative design can be created that functions with dual air pulses: one for the forward motion and the other one for the backward motion. This would require an additional air chamber, with flexible seal, as well as an additional air inlet. Vitrectomes based on this mechanical principle are already on the market. Although this would seem the simplest solution mechanically, this would complicate fabrication due to the additional air chambers from which support material needs to be removed. However, the performance of such a mechanism relies less on the spring characteristics of a 3D printed diaphragm than the current design, and therefore this could be a promising direction to further explore.

The outcomes of our research show the pros and cons of using 3D printing for fine-mechanical medical devices. We introduced a design methodology that can be used as a guideline for 3D printing of springs for various medical and industrial applications. Furthermore, we have explored possibilities for minimal-assembly 3D printing principles to circumvent challenges with tight tolerances and for dealing with support material in small internal cavities. Although there are still challenges to overcome, these are important steps towards real-life implementation of functional 3D printed devices in medicine.

## 5. Conclusions

In this study, we explored the possibility of creating a non-assembly vitrectome mechanism for eye surgery. The pneumatically actuated dual-diaphragm mechanism that we proposed to generate the linear motion required for this mechanism was successfully produced using multi-material PolyJet printing. To more efficiently produce the design, we considered the characteristics of the AM process by including features that allow the removal of support material, such as drainage holes and a screw connection. Although this adds an assembly step to the design, it provides access to remove and reduce support material and therefore decreases the total production time; therefore, we preferred these ‘smart assembly’ solutions over non-assembly. The two design directions for the dual diaphragm mechanism that were explored showed that the mechanism shows promise in terms of displacement and cutting force, although the cutting speed requirement could not be fulfilled. The viscoelastic properties of the materials influence the performance of the mechanism, which cannot be overcome by redesigning the diaphragms alone. The requirement of high stiffness for a suitable backward force leads to a contradictory, low speed response in the diaphragms. More research is needed to optimize the design for the required cutting speed. 

## Figures and Tables

**Table 1:** Ratios of the different material mixtures used for the diaphragms in the prototypes.

Name	Composition Vero	Composition Agilus30	Stiffness Indication
80Agilus20Vero	20%	80%	flexible
50Agilus50Vero	50%	50%	medium
20Agilus80Vero	80%	20%	stiff

**Table 2:** Results of the digital material diaphragms, showing the average backward cutting force, the average spring coefficient, and the average pressure required to displace the carrier by 0.8 mm. In addition, the fastest obtained forward and backward response for a displacement of 0.8 mm are given.

Prototype Name (Stiffness Indication)	Backward Cutting Force (*n* = 18)	Spring Coefficient (*n* = 18)	Pressure to Reach 0.8 mm Displacement (*n* = 2)	Fastest Forward Response 0.8 mm (*n* = 1)	Fastest Backward Response 0.8 mm (*n* = 1)
20Agilus80Vero (stiff)	8.9 ± 0.92 N	11.7 ± 1.53 N/mm	257 kPa	0.567 s	30.5 s
50Agilus50Vero (medium)	3.6 ± 0.27 N	5.2 ± 0.28 N/mm	171 kPa	0.156 s	9.7 s
80Agilus20Vero (flexible)	3.3 ± 0.16 N	4.5 ± 0.18 N/mm	156 kPa	0.110 s	9.2 s

**Table 3:** Properties of the ortho-planar spring designs used in the second test round.

Name	Thickness Spring	Material Spring	Thickness Membrane	Material Membrane
Z0.28	0.28 mm	100% Vero	0.50 mm	100% Agilus30
Z1.12	1.12 mm	100% Vero	0.50 mm	100% Agilus30
Z2.24	2.24 mm	100% Vero	0.50 mm	100% Agilus30

**Table 4:** Results of the spring diaphragm tests, showing the backward cutting force, spring coefficient, fastest forward response for 0.8 mm displacement, and fastest backward response for 0.8 mm displacement. If no values are given, the prototypes were unable to fulfill the entire 0.8 mm displacement due to leakages occurring before the test could be completed.

Name	Backward Cutting Force (*n* = 3)	Spring Coefficient (*n* = 3)	Fastest Forward Response 0.8 mm (*n* = 1)	Fastest Backward Response 0.8 mm (*n* = 1)
Z0.28	0.48 ± 0.48 N	0.6 ± 0.60 N/mm	-	-
Z1.12	4.00 ± 0.25 N	5.0 ± 0.32 N/mm	37.7 s	47.0 s
Z2.24	11.01 ± 0.49 N	13.8 ± 0.61 N/mm	3.1 s	-

**Table 5:** Fastest response times obtained for half of the 0.8 mm displacement.

Name	Fastest Response between 0 and 0.4 mm Forward (*n* = 1)	Fastest Response between 0.6 and 0.2 mm Backward (*n* = 1)
Z0.28	0.0019 s	0.4 s
Z1.12	0.0070 s	5.4 s
Z2.24	0.0056 s	2.5 s

## Data Availability

Data is contained within the article.
